# Abrupt increase in hydrogen diffusion on transition-metal surfaces during hydrogenation catalysis†

**DOI:** 10.1039/c6sc01249c

**Published:** 2016-04-18

**Authors:** Juan Simonovis, Francisco Zaera

**Affiliations:** a Department of Chemistry and UCR Center for Catalysis, University of California Riverside CA 92521 USA zaera@ucr.edu

## Abstract

A sharp increase in the rate of hydrogen isotope scrambling was identified during the hydrogenation of olefins with H_2_ + D_2_ mixtures on Pt(111) catalysts, which spectroscopic data suggest is due to a sudden increase in atomic hydrogen surface mobility because of a decrease in the size of the islands of the adsorbed hydrocarbons.

## Abstract 2

Hydrogen surface diffusion is initially hindered during olefin hydrogenation catalysis but abruptly increases at olefin pressures below a well-defined threshold.

## Graphical abstract

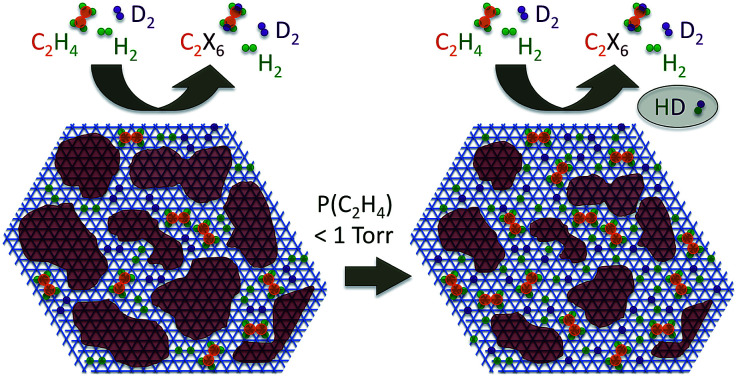

## Introduction

Thanks to their ability to readily dissociate molecular hydrogen, transition metals are used extensively to promote the hydrogenation of unsaturated bonds.^1,2^ The kinetics of H_2_ dissociation and recombination on late transition metals has been studied extensively using molecular beams and other modern surface-sensitive techniques, and determined to be fast even under mild conditions, namely, at low temperatures and pressures.^3–5^ However, virtually nothing is known about the behavior of H_2_ on metal surfaces under the conditions used for catalysis.^6–9^ Significant differences are expected, because such catalysis does not take place on the pristine metal but rather on surfaces covered with strongly-bonded carbonaceous deposits, alkylidynes in the case of olefins,^10–13^ as well as with reversibly-adsorbed reactants^7,14^ The kinetics of H_2_ adsorption–desorption on such surfaces are unknown, but expected to be much slower. In fact, they may perhaps limit the overall rate of the catalytic hydrogenations, given that the kinetics of those processes are typically first order in hydrogen pressure.^2,7,15^

In this report we discuss results from experiments aimed to probe those kinetics. Isotope labeling has been used to follow the kinetics of hydrogen adsorption, diffusion, and desorption during olefin hydrogenation catalysis. Specifically, gas mixtures composed of regular molecular hydrogen, molecular deuterium, and ethylene were reacted with the aid of a platinum single-crystal surface while following the kinetics of both ethane and HD formation. The key observation from this work is that the isotopes of hydrogen scramble at a rate that shows a highly non-linear transition during reaction. This abrupt switch in reaction kinetics is not accompanied by similar changes in alkane formation. With the aid of data from both mass spectrometry analysis of the gas phase and infrared adsorption spectroscopy characterization of the adsorbed species, we explain the observed behavior as a consequence of a subtle change in the size of the islands of the strongly adsorbed hydrocarbon fragments, in this case ethylidyne moieties, that form and are present on the surface during the catalysis. The unique kinetics of hydrogen isotope scrambling reported here helps explains the non-statistic distribution of isotopomers in some deuteration reactions, and could be general; it points to the importance of the heterogeneities of adsorbed layers in defining reaction kinetics on surfaces.

## Results

As stated above, in order to probe the relative rates of hydrogen adsorption and desorption *versus* olefin hydrogenation steps under catalytic conditions, we have measured the kinetics of HD and ethane formation simultaneously using C_2_H_4_ + H_2_ + D_2_ mixtures and a Pt(111) single crystal as the catalyst. These experiments were carried out in an instrument that combines surface-sensitive analytical techniques that operate under ultrahigh vacuum (UHV) conditions with an atmospheric-pressure batch reactor in order to be able to clean and fully characterize the surface and then perform the catalytic runs without exposing the solid to polluting external elements.^13,16,17^ The time evolution of the reactants and products was followed continuously using mass spectrometry detection while the nature of the species adsorbed on the Pt(111) surface was characterized *in situ* using reflection-absorption infrared spectroscopy (RAIRS).^18^

**Fig. 1:**
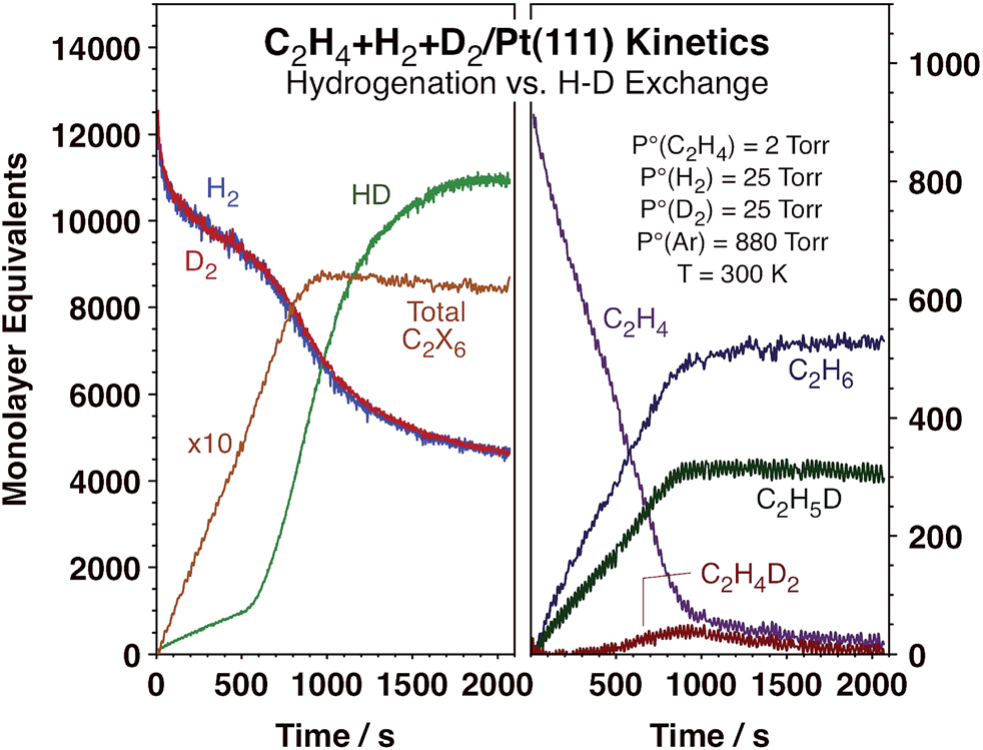
Kinetic data for the conversion of a mixture of 25 Torr H_2_, 25 Torr D_2_ and 2 Torr C_2_H_4_ promoted by a Pt(111) surface at 300 K. A sudden increase in HD production rate is seen around 500 s not matched by any significant changes in the rate of ethylene hydrogenation, which remains approximately constant until full conversion at approximately 1000 s. This non-linear change in HD production kinetics in the middle of the hydrogenation catalysis is the key observation from the present study.

Fig. 1 displays typical kinetic data from our experiments. Shown are the temporal evolution of the concentrations of the reactants, H_2_, D_2_, and C_2_H_4_, and the products, HD and ethane, in terms of monolayer equivalents (ML_eq_, molecules per Pt surface atoms). Several ethane isotopomers are produced, and the individual accumulation curves of the most relevant ones are also displayed in Fig. 1. An alternative report of the data in terms of turnover frequencies, TOFs (ML_eq_/s), obtained by numerical differentiation of the data in Fig. 1, is provided in Fig. S1 (ESI†). It is clearly seen in both figures that the conversion of C_2_H_4_ to C_2_H_6_ proceeds at an approximately constant rate until 100% conversion is reached in about 1000 s. On the other hand, the production of HD starts at a low steady rate but suddenly jumps after about 500 s of reaction, at which point its rate of formation increases by approximately one order of magnitude (to the values seen with H_2_ + D_2_ mixtures without any olefin in the reaction mixture, Fig. S2†). More accurately, the rate of overall ethane formation does increase by about ∼15% at the HD rate transition time (Fig. S1†), but the change is much more subtle than that seen in the HD curves, where the TOF transitions from approximately 1.5 s^−1^ to ∼16 s^−1^. It is this sudden and unexpected change in hydrogen isotope scrambling rate, which is not accompanied by any significant changes in either the rate or the isotope composition of the ethane production, that is the focus of our report.

The HD kinetic transition was seen to always occur after the C_2_H_4_ partial pressure reaches a threshold value of approximately 0.8 Torr (Fig. S3, ESI†), suggesting that such transition depends on the coverage of the adsorbed species produced by ethylene adsorption. It is worth emphasizing again that the change in the rate of accumulation of HD in the gas phase is quite abrupt, with the inflection point occurring over a change in ethylene partial pressure of less than 10% (and over changes in H_2_ and D_2_ partial pressures of less than 4%). Since the catalysis in these experiments has been carried out under conditions where ethylene adsorption is extensive and the coverage of the pi-bonded ethylene believed to be the intermediate for hydrogenation close to saturation, such small changes in ethylene partial pressure are expected to result in even more subtle variations in adsorbed ethylene coverages. These changes alone are not sufficient to explain the sudden increase in HD production around 500 s reported here.

**Fig. 2:**
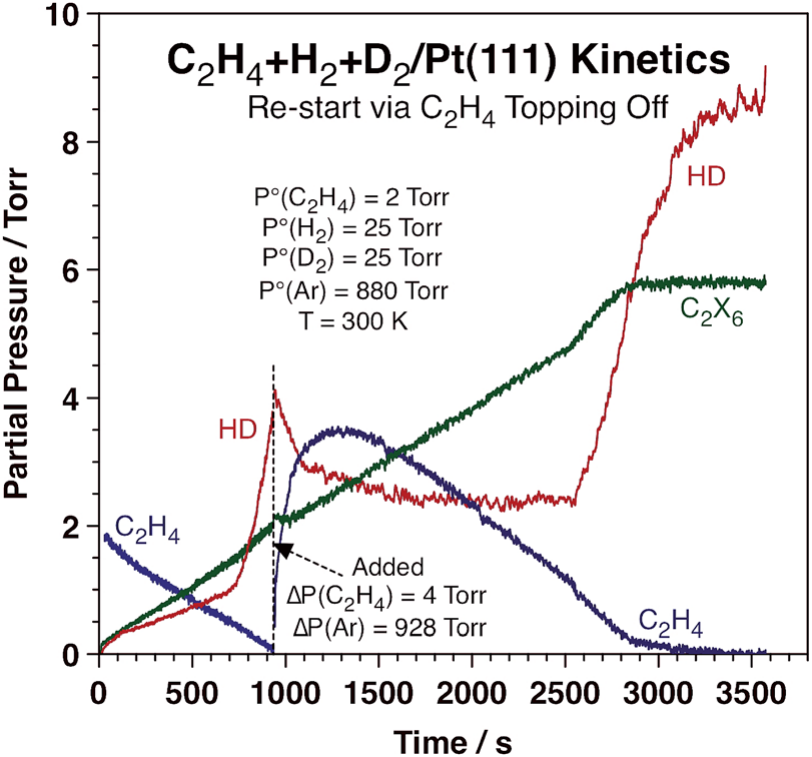
Kinetic data from an experiment where the partial pressure of ethylene was topped off after the HD production rate had already gone through the transition shown above. The net effect from this extra C_2_H_4_ is a switch back to the low-HD-production-rate regime until the partial pressure of ethylene is reduced again to below 0.8 Torr, at which point the HD accumulation curve shoots up again. This attests to the reversibility of the HD kinetic transition. No accompanying changes in the kinetics of ethane production were seen throughout the experiment.

It was also determined that the switchover in HD production rate is reversible. Typical data highlighting this fact are presented in Fig. 2, which corresponds to an experiment where extra ethylene was added to the reaction mixture after approximately 940 s of reaction, long after the sharp increase in HD production rate and close to the end of the conversion of the initial ethylene. It is clearly seen that the HD production is quenched immediately upon the addition of this extra ethylene (in fact, the concentration of HD in the gas phase decreases because of a dilution effect), and also that the rate of ethane production proceeds unaffected. The HD TOF switches back to the high-rate regime once the pressure of ethylene is lowered to the threshold value of ∼0.8 Torr, after 2560 s of reaction. Full conversion of the new ethylene to ethane is completed after ∼2850 s.

Our study of the temperature dependence of the kinetics of this process was not too informative, because the time at which the HD kinetics undergoes the indicated transition decreases rapidly with increasing temperature and is already too fast to be detectable by approximately 330 K (Fig. S4 in ESI†). What can be said from these data is that in the low-ethylene-pressure (high-time) regime the activation energy is comparable to the heat of adsorption reported for intermediate atomic hydrogen coverages on clean Pt(111) based on surface-science experiments.^19^ This is the regime that can be associated kinetically with hydrogen isotope scrambling on a clean platinum surface.

One viable explanation for the two HD production regimes reported above is that the reaction that produces the mixed-isotope product may occur on two different catalytic sites. Although we carried out the experiments on a Pt(111) single crystal specifically to minimize this potential complication, it is known that even such samples may have a small percentage of steps, kinks, and other types of defects. It has also been established that, on clean platinum surfaces, hydrogen scrambling is much faster on such defects.^4,20^ In order to investigate the possibility that defect sites on the surface may be driving the fast HD formation regime, additional kinetic experiments were carried out on surfaces where the defects were selectively blocked by sulfur atoms (by dosing small amounts of 1-propanethiol under UHV and decomposing the adsorbed species at 725 K).^21,22^ The data, provided in Fig. S5 (ESI†), clearly show that the addition of small amounts of sulfur to the surface does not poison the fast HD formation regime. Instead, the main effect is to reduce the initial rate of HD production. This, it should be emphasized, is not accompanied by any significant changes in the kinetics of production of ethane.

**Fig. 3:**
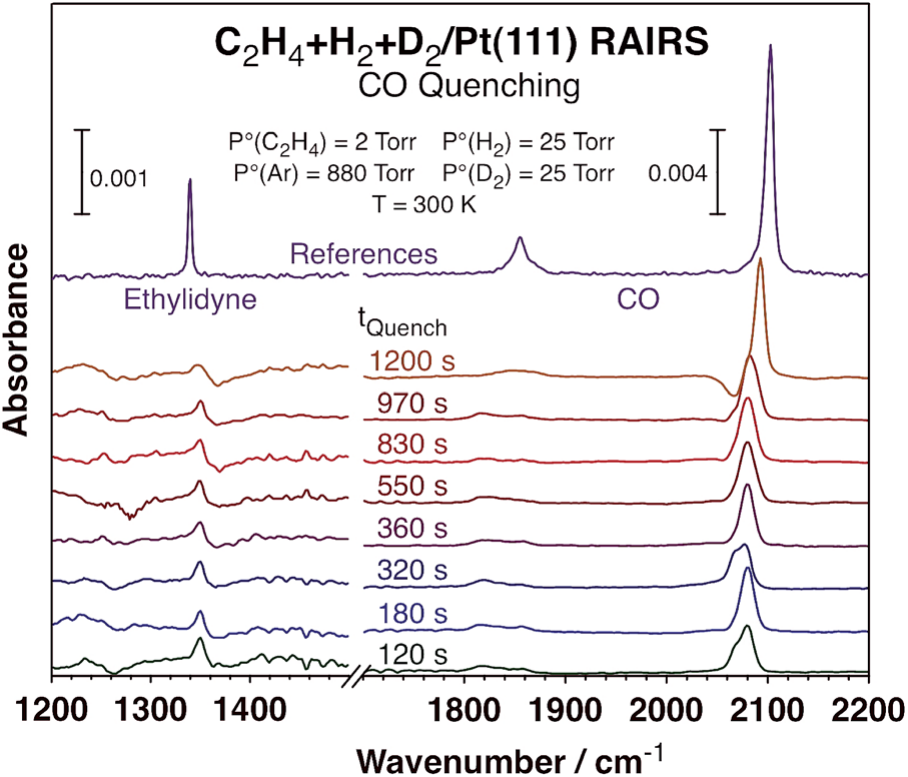
Reflection-absorption infrared spectra (RAIRS) from the Pt(111) surface after quenching the conversion of C_2_H_4_ + H_2_ + D_2_ mixtures by adding carbon monoxide to the gas at different reaction times. The peaks associated with CO (1800–1870 and 2080 cm^−1^) and ethylidyne (1350 cm^−1^) were used to estimate the evolution of the surface coverages of the metal sites and the carbonaceous deposits, respectively, as a function of reaction time (the data are reported in Fig. 4). The top (purple) traces are reference spectra from saturation layers of ethylidyne (left) and CO (right).

To better understand the kinetic changes reported here, *in situ* spectroscopic studies of the surface were carried out by using reflection-absorption infrared spectroscopy (RAIRS), which were combined with quenching of the reaction at different times by adding carbon monoxide to the reaction mixture^23^ in order to get a snapshot of the nature and coverage of the surface species during the course of the catalytic conversion.^24^ The infrared absorption spectra recorded after such quenching are displayed in Fig. 3. Clear signals are seen around 2080 cm^−1^ for the C–O stretching mode of carbon monoxide adsorbed on atop Pt sites, an indication that a significant fraction of the platinum surface remains exposed and available for reaction all throughout these catalytic runs (those are the sites capable of the CO uptake seen in these spectra). Moreover, the fact that the peaks are red-shifted with respect to what is seen for CO adsorption on clean Pt(111) suggests that the Pt open sites, where the CO adsorbs, are intermingled with other adsorbed species, ethylidyne in this case. The peak shapes and peak positions vary somewhat among the different spectra, but we believe that those fluctuations may not be significant but rather a consequence of experimental artifacts, given the difficulty to exactly reproduce the nature of the surface during catalytic reactions and because these spectra need to be ratioed against appropriate background traces. The most important information to be extracted from these data is the coverages of open Pt sites available for reaction *versus* reaction time, as discussed next, and those data are not sensitive to the position or shape of these peaks.

The areas under the main peaks were analyzed according to previous literature reports^25^ in order to quantify the coverages of the respective species and correlate them to the extent of reaction, as mentioned above. Specifically, the areas under the peaks associated with CO (1800–1870 and 2080 cm^−1^) and ethylidyne (1350 cm^−1^) were calibrated to provide values for the surface coverages of those adsorbed species (the CO coverage is used here as a measure of the number of platinum sites available for reaction). Particular attention was placed on calibrating the signals for CO, since those are well known to deviate from linearity at high coverages because of strong intermolecular dipole–dipole interactions. Both ourselves^26^ and other groups^27,28^ have clearly shown this effect under ultrahigh vacuum (UHV) conditions and adjusted for it. In this case the CO signals are measured under atmospheric conditions and on surfaces covered with other species (alkylidyne) and that may render previous IR signal calibrations inapplicable here, but if anything the deviations from non-linearity may be less marked because of the dilution effect (the CO peaks are indeed red-shifted in Fig. 3 from their values under vacuum, as mentioned above and expected if less dipole–dipole interactions are in play). Regardless, the coverage variations seen here are small enough for this effect not to be critical. Additionally, the trends observed with CO roughly mirror the ones extracted from the ethylidyne peak, which does not suffer from the same limitation. Finally, the arguments discussed below rely on relative trends, not absolute coverage values, and those are less affected by these signal calibration.

**Fig. 4:**
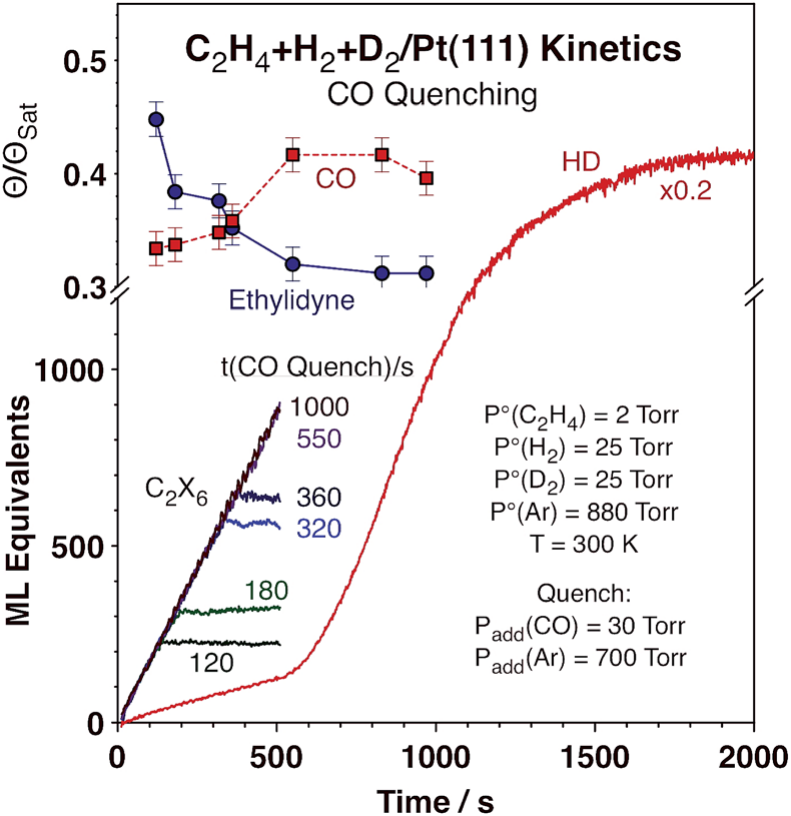
Correlation between the kinetics of ethylene hydrogenation and HD formation and the surface coverages of ethylidyne and carbon monoxide after quenching the reaction with CO at various times. The ethane accumulation curves provided in the lower left corner attest to the effectiveness of CO in suppressing all catalytic activity, whereas the surface coverage data in the upper left corner indicate a reduction in ethylidyne coverage and an opening of more sites around the time where the HD production rate (red trace in main frame, without CO quenching) changes from slow to fast.

A summary of the results from the titration experiments in terms of coverages of the adsorbed species *versus* reaction time is shown in Fig. 4. While the kinetic data attest to the effectiveness of the added CO in suppressing all catalytic activity (Fig. 4 only shows the data for ethane formation, but the halting of the HD formation with CO addition was also evident), the information extracted from the infrared absorption spectra indicates that there is a decrease in ethylidyne coverage, and a concurrent increase in CO coverage (which, as said before, reflects the fraction of the metal surface exposed for reversible adsorption and reaction during catalysis) with increasing reaction time, with a more visible jump in those quantities around the time when the HD production kinetics switches from slow to high. It should be indicated that the observed changes are somewhat limited, amounting to only about 20% of their total values, and that the measurements of surface coverages carry significant error bars. Nevertheless, they do seem to correlate reasonably well with the kinetic switchover discussed in this report.

**Fig. 5:**
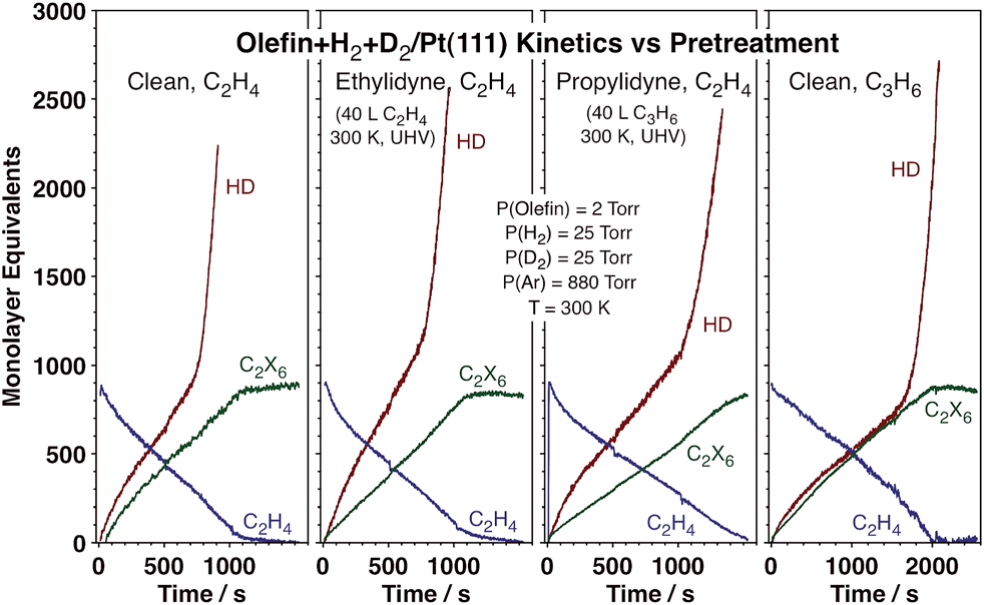
Kinetic data for the hydrogenation of either ethylene (first three frames) or propylene (last frame) with H_2_ + D_2_ mixtures on Pt(111) surfaces, clean (1st and 4th frames) or pre-modified with saturation layers of ethylidyne (2nd frame) or propylidyne (3rd frame). The absolute values of the reaction rates change somewhat across this series, but the qualitative observation of a sharp HD rate switch during olefin hydrogenation is evident in all cases.

The data reported above point to the role that the strongly bonded carbonaceous deposits, that is, the ethylidyne layer that forms on the surface during reaction, play in affecting the kinetics of hydrogen scrambling. In order to test that effect further, contrasting kinetic experiments were carried out on Pt(111) surfaces precovered with different alkylidyne layers (ethylidyne or propylidyne) as well as for the conversion of propylene instead of ethylene (on the initially clean metal). The data, reported in Fig. 5, attest to the generality of the HD switching kinetics. Predosing the surface with ethylidyne results in no kinetic changes at all, not surprisingly because an ethylidyne layer forms on the clean Pt within the first seconds of exposure to the ethylene + hydrogen reaction mixture anyway. Replacing the ethylidyne layer with propylidyne slows down all reactions, but still allows for the HD rate transition at approximately the same threshold ethylene pressure. Finally, all initial reactions are slower with propylene, but a transition to fast HD production is still seen before the olefin hydrogenation (which is not affected) is over. Overall, the changes in the kinetics of the hydrogenation and isotope scrambling reactions seen upon varying the nature of the carbonaceous deposits or the gas-phase olefin are quantitative rather than qualitative, and the HD rate switchover midway throughout the olefin conversion general.

## Discussion

The emulation of steady-state olefin hydrogenation catalysis under vacuum has by and large eluded the surface-science community,^29–31^ even though catalytic hydrogenations under atmospheric pressures are relatively facile reactions. Also, the kinetics of most hydrogenation catalytic reactions are approximately first order in hydrogen and zero or negative order in the organic reactant, indicating that the rate limiting step involves hydrogen atoms,^2^ but they also display quite low reaction probabilities, on the order of one conversion per million collisions of the reactants with the catalytic surface;^7^ this is in contrast to the almost-unity sticking coefficients measured for hydrogen under vacuum.^4^ If the kinetics of catalytic hydrogenations are limited by the availability of hydrogen on the surface and hydrogen adsorbs readily on metal surfaces, why don't these reactions display higher reaction probabilities?

The answer to that question lies, at least in part, in the fact that catalytic hydrogenations do not occur on clean metal surfaces but rather on surfaces covered with hydrocarbons. For the case of small olefins, it has been shown that those species include alkylidynes, which are formed immediately upon exposure of the transition metal surfaces to the reactants^32^ and remain strongly bonded and can only be removed slowly throughout the catalytic process.^7,10,12^ In addition, under catalytic conditions, the gas-phase reactants reach an equilibrium with high coverages of adsorbates.^33^ Both irreversibly and reversibly adsorbed hydrocarbons block the sites needed for the dissociative adsorption of hydrogen on the surface of the catalyst. Early catalytic studies with mixtures of olefins and either H_2_ + D_2_ or *ortho*-H_2_ + *para*-H_2_ (ref. 34 and 35) led to the conclusion that hydrogen scrambling under catalytic hydrogenation conditions is in fact quite slow. The experiments reported here were initially designed to identify the transition from the high-probability non-catalytic hydrogenation regime seen under vacuum to the low-probability steady-state process associated with typical hydrogenation catalysis.^31^

In this context, the sharp transition in HD production rate that takes place during olefin hydrogenations could be interpreted as the result of a sudden change in hydrogen and deuterium surface coverages (θ_X_, X = H or D). However, since neither the rate of ethylene hydrogenation nor the isotope distribution of the ethanes produced is affected in any significant way during the transition, that does not appear to be a viable explanation. The main difference between the hydrogen scrambling and olefin hydrogenation kinetics is that while the latter displays a linear dependence on θ_X_,^29^ the former requires two of such atoms and therefore should show a higher rate-law θ_X_ order. This suggests that it is the scrambling of the hydrogen atoms, that is, their mobility and recombination on the surface, that limits the formation of HD in the initial stages of the reaction. It is also quite likely that the kinetics of atomic hydrogen scrambling on the surface is complex, because the surface is covered with hydrocarbons, which may hinder atomic hydrogen diffusion. A sudden change in hydrogen mobility, due to a change in the hydrocarbon layer on the surface, is what must cause the abrupt switch in HD production observed with decreasing olefin pressure.

The non-linear nature of the kinetic change in HD production kinetics also points to a critical sudden change on the surface occurring at the threshold ethylene pressure identified in our experiments. This may be understood on the basis of the nature of the adsorbed alkylidyne layer, which has been shown not to be uniform but rather to form islands on the surface.^36^ We propose a model in which these islands are large enough during the early stages of the reaction to coalesce and create isolated areas of uncovered platinum in between where H_2_ and D_2_ can adsorb and dissociate but not mix. As the hydrogenation proceeds, the olefin pressure decreases and the alkylidyne islands become smaller (because of their partial removal from the surface, given that ethylidyne deposition depends on ethylene pressure but its removal does not),^13,25,31,33^ to the point that they may no longer overlap with each other. That results in the opening of pathways on the surface between the previously isolated patches of bare metal, allowing the adsorbed hydrogen and deuterium atoms to travel longer distances and to scramble and recombine as HD. Presumably, this transition occurs over the narrow range of ethylidyne coverages identified in Fig. 4.

It would be desirable to be able to develop a microkinetic model that can reproduce the observations reported above. Unfortunately, our attempts to do that have all been unsuccessful so far. We found that kinetic calculations based on a mean-field treatment of the rates of feasible mechanistic steps proposed for this catalysis do not have the ability to reproduce sharp, highly non-lineal kinetics occurring at intermediate times during reaction. This is true even with kinetic models where more than one type of adsorption site and/or one or more rate limiting steps are proposed, as has been done to explain other kinetic features of the catalytic hydrogenation of ethylene.^37,38^ The inclusion of either autocatalytic^39^ or “surface explosion”^40,41^ steps, which do display non-linear kinetics, were not helpful in this case either, because the transition that we report occurs at an intermediate time in the reaction, with intermediate values of the partial pressures of all reactants. We believe that the phenomenon we report is due to inhomogeneities on the surface, *i.e.*, the presence of ethylidyne islands, leading to different local kinetic behavior, and that this is not well captured by mean-field kinetic calculations. It may perhaps be possible to reproduce our kinetic results using spatially-resolved Monte Carlo simulations,^42–44^ but that is a complex task outside the objectives of this work.

The sudden change in HD kinetics reported here is therefore not associated with a transition in hydrogenation rates but rather with a change in surface mobility of adsorbed hydrogen. This limitation in hydrogen surface diffusion is an important observation that explains the non-statistical isotopomer distributions seen in some olefin hydrogenation reactions.^45^ It also highlights a potentially general effect induced by the non-homogeneity of the distribution of adsorbed species that may affect the kinetics of many chemical reactions on surfaces. Further studies are needed to test both the generality of the behavior and its implications for catalysis.

## Experimental

The experiments reported here were carried out in a homemade ultrahigh vacuum (UHV) instrument equipped with an ion sputtering gun for sample cleaning and a mass spectrometer for gas identification and partial pressure measurements.^13,18^ The latter is interfaced to a computer, and programmed to be able to follow the time evolution of up to 15 different masses in a single experiment. The chamber also contains a sealable cell capable of pressurization, used as the batch reactor where the catalytic reactions are carried out. A Fourier-transform infrared absorption spectrometer is set up in reflection-absorption (RAIRS) mode for the *in situ* characterization of the surface during gas exposures, simultaneously as the kinetics of the reactions are followed continuously by mass spectrometry by taking advantage of a controlled small leak of the gases from inside the cell into the UHV chamber. The catalyst used consists of a Pt single crystal, a disk ∼1 cm in diameter and ∼1 mm in thickness, cut and polished to expose its (111) facet.

In a typical experiment, the Pt(111) crystal was first cleaned in the UHV chamber by a combination of sputtering, oxygen treatment, and annealing. The sample was transferred to the side chamber, where the sealable atmospheric-pressure cell is located, and background RAIRS data were acquired with both s- and p-polarized light. The Pt was exposed to the desired gases if a pretreatment was required (typically ethylene or propylene but also 1-propanethiol in Fig. S5†), a new set of RAIRS acquired, the high-pressure cell closed, and yet another pair of RAIRS traces recorded. The crystal was then heated to the desired temperature, and the reaction mixture, premixed in a side gas manifold, let into the reaction volume. The gas was recirculated within the close loop used as the batch reactor using a bellows pump, and its composition was monitored continuously by mass spectrometry until the reaction reached completion, after which the volume of the reactor was pumped with a turbomolecular pump and the cell retracted to expose the crystal back to the UHV environment. RAIRS traces were taken during the course of the catalytic conversion as well as immediately before and right after opening the cell.

The mass spectrometer in the kinetic experiments was set to record signals for all masses in the 2–4 and 26–36 amu ranges in every run in order to obtain information about the time evolution of all the relevant reactants and products, namely, H_2_, HD, D_2_, and all the isotopomers of ethylene and ethane (a similar approach was followed for the propylene experiments reported in Fig. 5 and S2†). Partial pressures for each compound were calculated from the mass spectrometer signals by following a matrix-based deconvolution procedure described in detail in previous publications,^46,47^ after input of the cracking patterns of all the individual molecules. For the case of hydrogen, this process is relatively simple, requiring only the subtraction of the contributions from HD and D_2_ to the 2 amu signal. For the ethylenes and ethanes, on the other hand, multiple interferences need to be taken into account, but our analytical procedure is capable of fully addressing this issue.

## Conclusions

The studies reported here focused on the characterization of the adsorption–desorption kinetics of hydrogen during catalytic hydrogenation reactions. To this end, isotope labeling was employed to follow the scrambling of hydrogen atoms on the surface. Mixtures of H_2_ + D_2_ + C_2_H_4_ were reacted at room temperature and atmospheric pressures in the presence of a Pt(111) single-crystal surface, which was used as the catalyst. The olefin hydrogenates readily to its corresponding alkane under these conditions, following simple kinetics similar to those reported in the past for both single-crystal and more realistic supported catalysts. A distribution of ethane isotopomers is produced, again in line with what has been seen with similar systems in the past.

The surprising new observation here is that the kinetics of hydrogen isotope scrambling, which we followed by the detection of the HD product, shows a sharp transition between two very different kinetic regimes, in with the value of the rate of its production jumps by about one order of magnitude. This happens during the course of the olefin hydrogenation, in our case when the partial pressure of ethylene reaches a value of approximately 0.8 Torr, and it is not accompanied by any other large kinetic changes. A concurrent reduction in the coverage of ethylidyne on the surface was identified by infrared absorption spectroscopy. These are strongly-bonded hydrocarbon fragments that form and remain on the surface during the catalytic hydrogenation, and we speculate that they may influence the diffusion of adsorbed atomic hydrogen and impair their scrambling before recombination. Since such carbonaceous deposits tend to form islands on the surface of the catalyst, the drop in their coverage seen at intermediate reaction times may lead to the opening of new channels for the hydrogen to diffuse and recombine with other hydrogen/deuterium atoms at previously isolated adsorption sites. The critical kinetic switch may occur at the time when the ethylidyne islands stop touching each other, which may be the reason for the blocking of the hydrogen diffusion between separate isolated Pt site regions, and when the metal sites become all accessible.

## Supplementary Material

SC-007-C6SC01249C-s001
