# Supplementary material for: Abrupt increase in hydrogen diffusion on transition-metal surfaces during hydrogenation catalysis
Source: Chem Sci. 2016 Apr 18;7(7):4660–6. doi: 10.1039/c6sc01249c (PMC6013773; doi:10.1039/c6sc01249c)
Supplement: SC-007-C6SC01249C-s001 [file SC-007-C6SC01249C-s001.pdf]

Electronic Supplementary Material (ESI) for Chemical Science.
This journal is © The Royal Society of Chemistry 2016

Abrupt increase in hydrogen diffusion on transition-metal surfaces during hydrogenation catalysis

Juan Simonovis and Francisco Zaera*

Department of Chemistry and UCR Center for Catalysis, University of California, Riverside, CA 92521, USA

Email: zaera@ucr.edu

Supplementary Information

Figure S1. Kinetic data in the form of TOF versus time for a typical C_2H_4+H_2+D_2/Pt(111) run.

Figure S2. Kinetic data for HD formation with versus without olefin in the reaction mixture.

Figure S3. P(HD) vs. P(C_2H_4) for several runs starting with different initial ethylene pressures.

Figure S4. HD TON versus time for reactions carried at different temperatures.

Figure S5 Kinetic data as a function of sulfur predose.

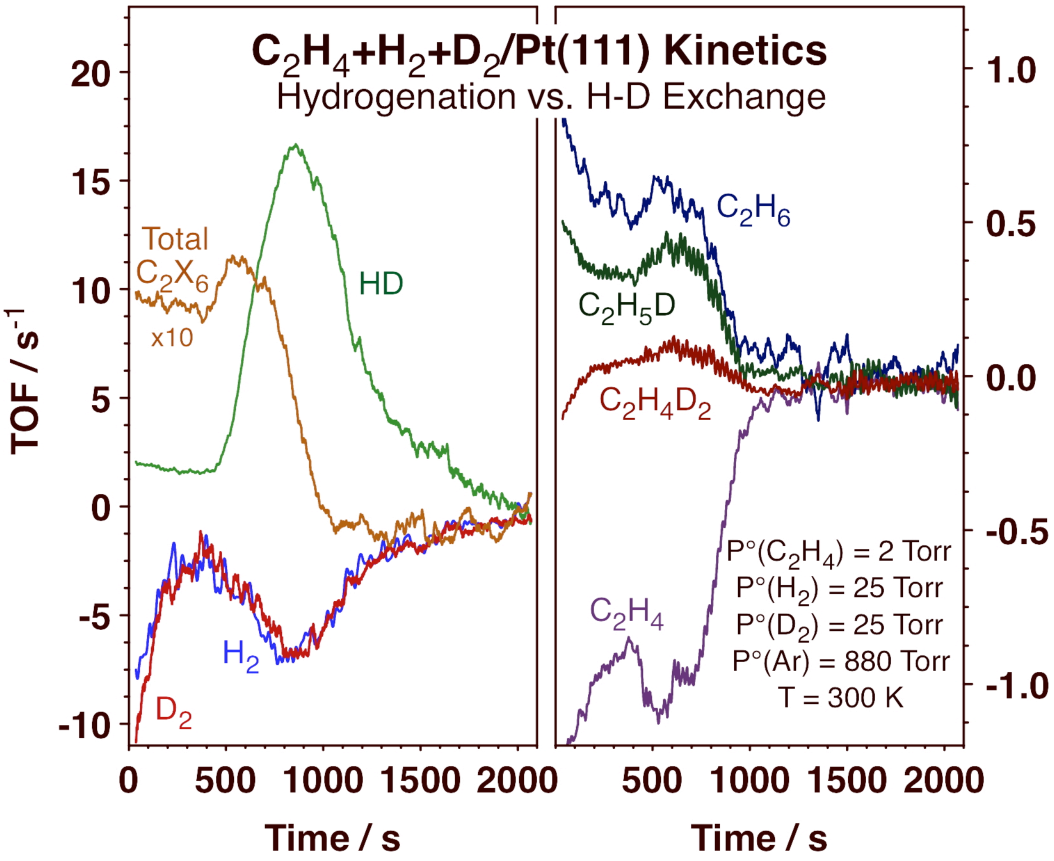

Figure S1

Turnover frequencies (TOF = ML_{eq}/s) for the conversion of a mixture of 25 Torr H_2, 25 Torr D_2, and 2 Torr C_2H_4 promoted by a Pt(111) single-crystal surface at 300 K, calculated via numeric differentiation of the data in Figure 1. Particularly noteworthy is the sudden increase in HD production rate that occurs around 500 s. By contrast, the time evolution of the TOF for ethylene conversion remains approximately constant (within ~15%) until full conversion, which is reached after approximately 1000 s.

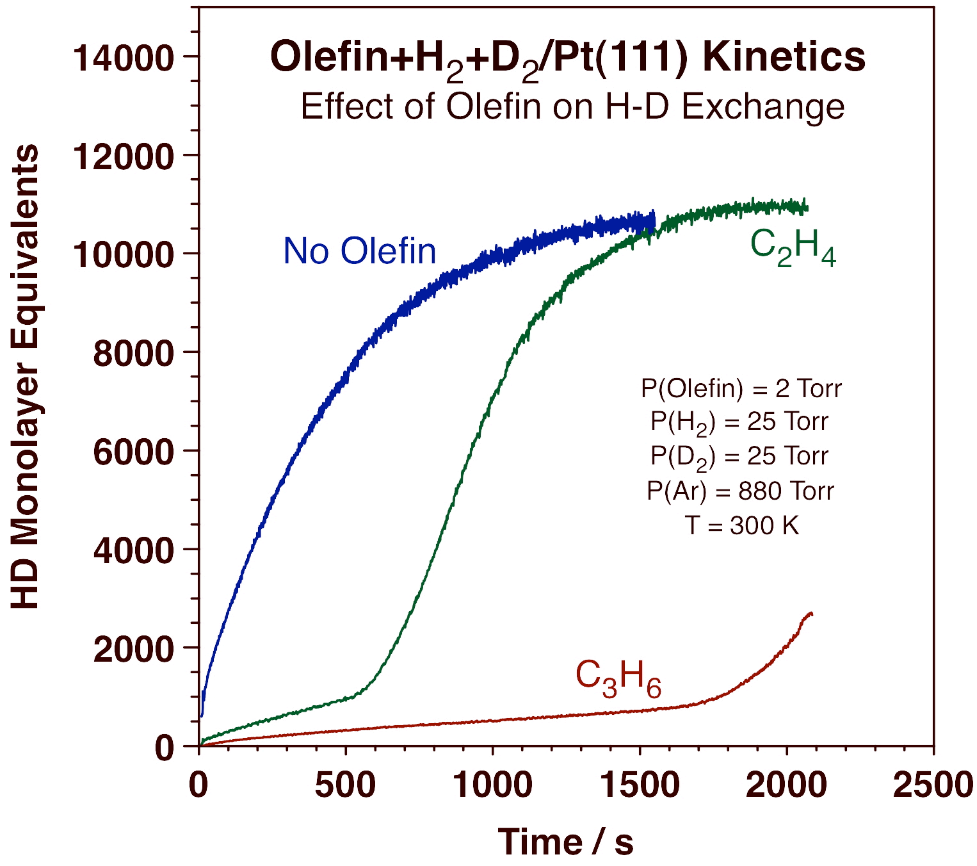

Figure S2

HD accumulation as a function of time, in experiments with a pure H_2 + D_2 mixture and where olefins (ethylene or propylene) were added. These data show how the second, fast HD production regime is similar to that seen on clean platinum. Although the main focus of this report is on the effect of ethylene on the rate of HD formation, the data for propylene has also been included to illustrate the generality of our conclusions.

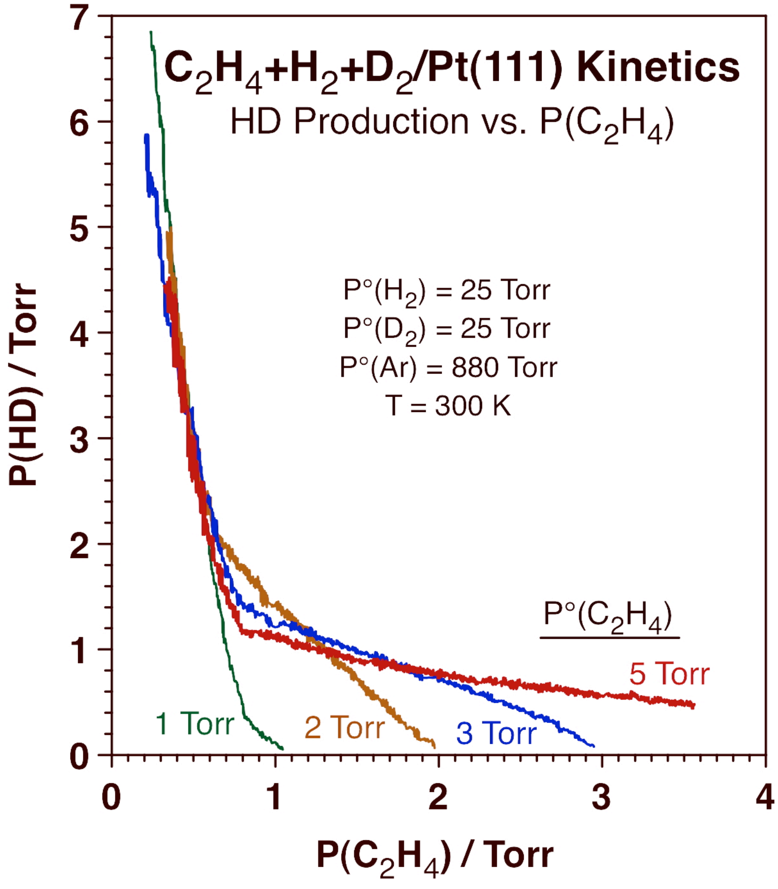

Figure S3.

HD production kinetics in the form of HD partial pressure versus C_2H_4 partial pressure for four experiments starting with varying initial ethylene pressures (all other conditions were kept the same). In this plot, the reaction proceeds from right (high $P(C_2H_4)$ to left (low $P(C_2H_4)$). The sharp kinetic transition discussed in this report is always seen at around $P(C_2H_4) \sim 0.8$ Torr, regardless of the initial pressure used. This way of presenting the data also highlights the narrow range of ethylene pressures over which the inflection point in the HD accumulation curves take place.

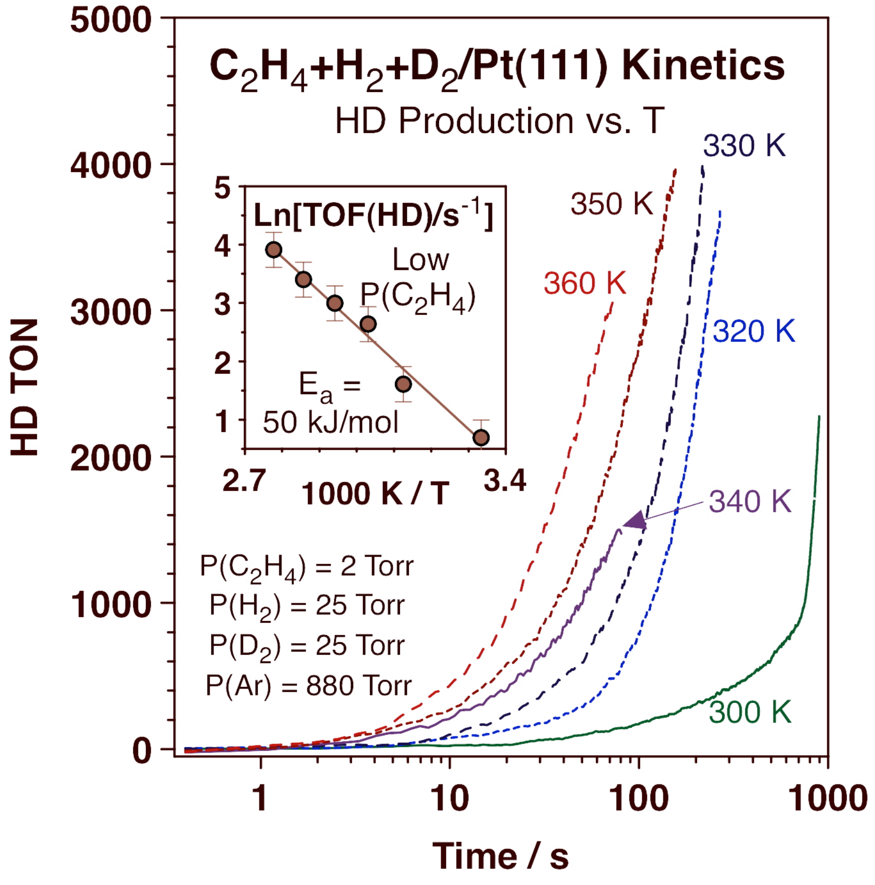

Figure S4

HD accumulation versus time (plotted in a logarithmic scale to better highlight the changes seen) in experiments carried out at different temperatures between 300 and 360 K. The HD kinetic transition subject of this report occurs at earlier times with increasing temperature, and is not measurable by ~ 340 K. The inset shows an Arrhenius plot of the TOFs estimated for the low-ethylene-pressure (long-times) regime. The activation energy in that regime is comparable to the heat of adsorption reported for intermediate H coverages on clean Pt(111) based on surface-science experiments.

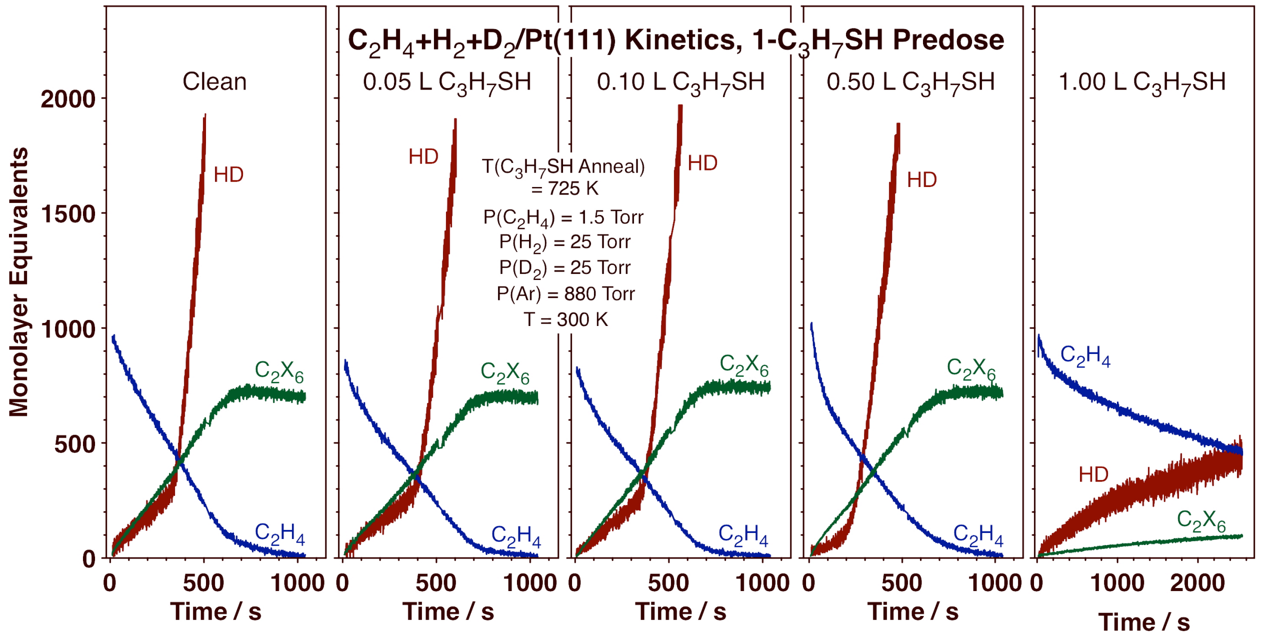

Figure S5

Kinetic data for the conversion of C_2H_4 + H_2 + D_2 mixtures on Pt(111) surfaces predosed with various amounts of sulfur, deposited via exposure to fixed amounts of 1-propanethiol under UHV followed by annealing to 725 K. At low S coverages the main effect observed is a slower initial rate of HD production. Higher sulfur coverages lead to non-selective surface poisoning (last panel).
